# Direct growth of single-crystalline III–V semiconductors on amorphous substrates

**DOI:** 10.1038/ncomms10502

**Published:** 2016-01-27

**Authors:** Kevin Chen, Rehan Kapadia, Audrey Harker, Sujay Desai, Jeong Seuk Kang, Steven Chuang, Mahmut Tosun, Carolin M. Sutter-Fella, Michael Tsang, Yuping Zeng, Daisuke Kiriya, Jubin Hazra, Surabhi Rao Madhvapathy, Mark Hettick, Yu-Ze Chen, James Mastandrea, Matin Amani, Stefano Cabrini, Yu-Lun Chueh, Joel W. Ager III, Daryl C. Chrzan, Ali Javey

**Affiliations:** 1 Department of Electrical Engineering and Computer Sciences, University of California, Berkeley, California 94720, USA; 2 Materials Sciences Division, Lawrence Berkeley National Laboratory, Berkeley, California 94720, USA; 3 Department of Chemical Engineering, University of California, Berkeley, California 94720, USA; 4 Department of Electrical Engineering, University of Southern California, Los Angeles, California 90089, USA; 5 Department of Materials Science and Engineering, National Tsing Hua University, Hsinchu 30013, Taiwan; 6 Department of Materials Science and Engineering, University of California, Berkeley, California 94720, USA; 7 Molecular Foundry, Lawrence Berkeley National Laboratory, Berkeley, 94720 California, USA

## Abstract

The III–V compound semiconductors exhibit superb electronic and optoelectronic properties. Traditionally, closely lattice-matched epitaxial substrates have been required for the growth of high-quality single-crystal III–V thin films and patterned microstructures. To remove this materials constraint, here we introduce a growth mode that enables direct writing of single-crystalline III–V's on amorphous substrates, thus further expanding their utility for various applications. The process utilizes templated liquid-phase crystal growth that results in user-tunable, patterned micro and nanostructures of single-crystalline III–V's of up to tens of micrometres in lateral dimensions. InP is chosen as a model material system owing to its technological importance. The patterned InP single crystals are configured as high-performance transistors and photodetectors directly on amorphous SiO_2_ growth substrates, with performance matching state-of-the-art epitaxially grown devices. The work presents an important advance towards universal integration of III–V's on application-specific substrates by direct growth.

## Abstract (web summary)

Growth of high-quality III–V semiconductors for electronics and optoelectronics usually requires an atomic-lattice matched substrate. Here, the authors use templated liquid-phase crystal growth to create single-crystalline III–V material up to ten micrometres across on an amorphous substrate.

## Graphical abstract

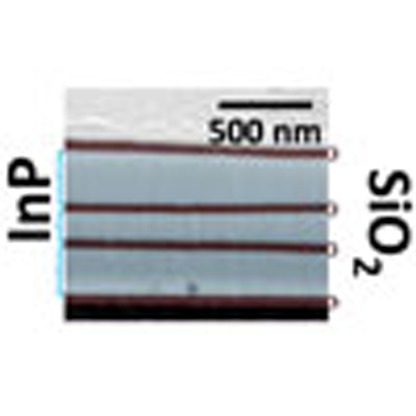

Owing to their high-electron mobilities and direct band gaps, III–V compound semiconductors are ideal for many electronic and optoelectronic applications such as high-performance transistors^1^,^2^,^3^, photovoltaics^4^,^5^, LEDs^6^ and photodetectors^7^. The development of epitaxial growth techniques such as molecular beam epitaxy^8^, metal-organic chemical vapour deposition (MOCVD)^9^ and pulsed laser deposition^10^ have enabled the growth of single-crystalline III–V thin films with excellent performance for device applications. In order to obtain such high-quality single-crystalline thin films, the growth must be done on a closely lattice-matched substrate^11^. The growth of single crystals onto amorphous substrates would further enable new applications, such as providing a simplified pathway for heterogeneous integration of III–V devices onto application-specific substrates. However, deterministic synthesis of single-crystalline semiconductors on amorphous substrates presents a fundamental challenge in the field of materials science—one that arises from the thermodynamics and kinetics of nucleation and crystal growth. Specifically, the slow kinetics governing coalescence of two grains into a single grain dictates that any single-crystalline structure on an amorphous substrate must be grown from a single nucleus. Thus, for single-crystalline growth, the first nucleus that forms must grow to fill the desired volume before another nucleus is formed. Within most growth approaches, the relative nucleation and growth rates are difficult to control, and the maximum grain size attainable is often on the order of the material thickness, resulting in nanocrystalline structures for nanoscale thickness materials^12^.

Because of the tremendous technology-driven need for single-crystalline semiconductors on amorphous substrates, a number of synthesis techniques have been explored in recent years. These approaches include (i) epitaxial growth of thin films on single-crystalline substrates followed by selective layer transfer to a desired substrate^2^,^4^, (ii) vapour–liquid–solid^13^,^14^,^15^,^16^,^17^, vapour–solid^18^,^19^ or aerosol-based^20^ nanowire growth. For GaN, in particular, the usage of ‘pre-orienting' layers to conduct local hetero-epitaxy has also been demonstrated^21^,^22^. While such approaches have resulted in broadening the scope and functionality of various electronic materials with unique properties, direct growth of single-crystalline semiconductors with ‘user-defined' geometries and dimensions on amorphous substrates has yet to be demonstrated. Such an approach would offer major advantages in terms of compatibility with traditional device processing technology, scalability and processing cost. In addition, it would provide a direct pathway to three-dimensional integration of electronic materials and devices with appreciable levels of complexity.

Here, we introduce templated liquid-phase (TLP) crystal growth (Fig. 1a) as a synthetic approach for growth of high-performance, nano- and micro-scale single-crystalline compound semiconductors with user-defined geometries on arbitrary substrates. Indium phosphide is chosen as a model III–V material system owing to its importance in a wide variety of fields, from high-speed electronics to lasers and photovoltaics^5^,^7^,^13^,^23^. Thermally grown SiO_2_ and glass are selected as examples of amorphous materials on which TLP crystal growth can be performed.

## Results

### TLP growth and crystal quality

For TLP growth of InP, indium metal is first lithographically patterned onto a Si/SiO_2_ or glass substrate with a thin (1–10 nm) MoO_x_ nucleation layer, and subsequently encapsulated by evaporated SiO_x_ (Supplementary Fig. 1). Growth is carried out in a low-pressure furnace at 500–535 °C in the presence of phosphine (PH_3_) and H_2_. At the growth temperature, In is transformed into the liquid phase, but remains mechanically confined by the SiO_x_ template. Phosphorous diffuses through the SiO_x_ cap, and supersaturates the liquid In, precipitating out in the form of an InP nucleus. The key feature of this growth mode is that a phosphorous depletion zone forms around each growing nucleus, preventing further nucleation. Previously, we have shown that for continuous In thin films, this depletion zone can be on the order of hundreds of μm, leading to ultra-large grain sizes^24^,^25^. Here, we show that through pre-patterning the indium in mechanically confined templates such that the phosphorous depletion zone from the first nucleus occupies the entire template, ‘single crystalline' InP growth in user-defined geometries can be achieved.

**Figure 1:**
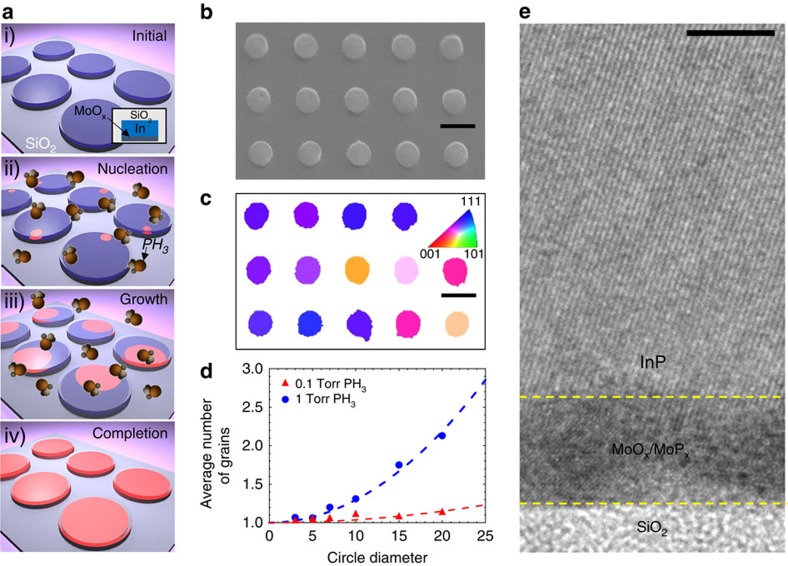
Growth mechanism of single-crystalline InP. (**a**) Schematic of the process flow for TLP crystal growth. (**b**) SEM images of an array of 7 μm InP circles and (**c**) their corresponding EBSD maps. Scale bar, 10 μm. (**d**) The average number of grains per circle, measured via EBSD versus the circle diameter, showing a quadratic dependence. (**e**) TEM cross-sectional image of a portion of a patterned InP thin film showing the well-defined InP lattice on top of a MoO_x_/MoP_x_ layer on amorphous SiO_2_. Scale bar, 5 nm.

Scanning electron microscope (SEM) images of the InP crystal arrays shaped into circles, rings and squares grown via TLP crystal growth are shown in Fig. 1b and Supplementary Fig. 2. Critically, the original In template geometry is maintained after growth, allowing for deterministic shape control of InP crystals. The stoichiometry of the films is confirmed by electron dispersive spectroscopy (EDS) to be 1:1 In:P. X-ray diffraction spectroscopy on an array of InP circles (Supplementary Fig. 3) displays only peaks corresponding to zincblende InP, indicating that all of the In has converted to InP. The crystallinity of the InP patterns with lateral dimensions of ∼5–7 μm is confirmed via electron backscatter diffraction (EBSD) mapping, showing that excluding twinning, each individual pattern is a single crystal but with different crystal orientations (Fig. 1c, Supplementary Fig. 2, Methods section, Supplementary Note 1). In addition, from the orientation distribution obtained from EBSD, it can be seen that there is a preferential texturing of the growth in the (1 0 *n*) direction , where *n* ranges between 1 and 2 (Supplementary Fig. 4).

To study the effect of growth conditions and InP feature size on the number of grains, InP circles with diameters varying from 3 to 20 μm were patterned using TLP crystal growth at two different PH_3_ partial pressures. From Fig. 1d and Supplementary Fig. 5, it can be seen that the number of grains per circle increases according to a quadratic relation with the circle diameter, *d*, which can be fit by the equation:


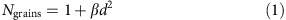


*N*_grains_ is the average number of grains per circle and *β* is a proportionality factor that takes into account growth parameters such as P flux, the geometry associated with nucleation and the resulting average nucleation rate (Supplementary Fig. 6, Supplementary Note 2). As expected from our model, there is also a strong dependence of the number of grains on the PH_3_ partial pressure. As the PH_3_ partial pressure is lowered, the P flux into the liquid decreases, resulting in a reduced nucleation rate and larger P depletion zones (Supplementary Note 2). This is reflected in a drop in *β* from 3 × 10^−3^ to 4 × 10^−4^ as the PH_3_ partial pressure is reduced from 1 to 0.1 Torr, allowing the average number of grains per feature to be maintained at near unity even for diameters as large as 20 μm.

Transmission electron microscopy (TEM) was used to examine the crystallinity of the InP patterns. From the cross-sectional TEM image of an InP sample shown in Fig. 1e, it can be seen that a crystalline InP lattice sits on top of the amorphous SiO_2_ substrate with a thin MoO_x_/MoP_x_ nucleation layer in between, clearly showing the non-epitaxial nature of TLP crystal growth.

**Figure 2:**
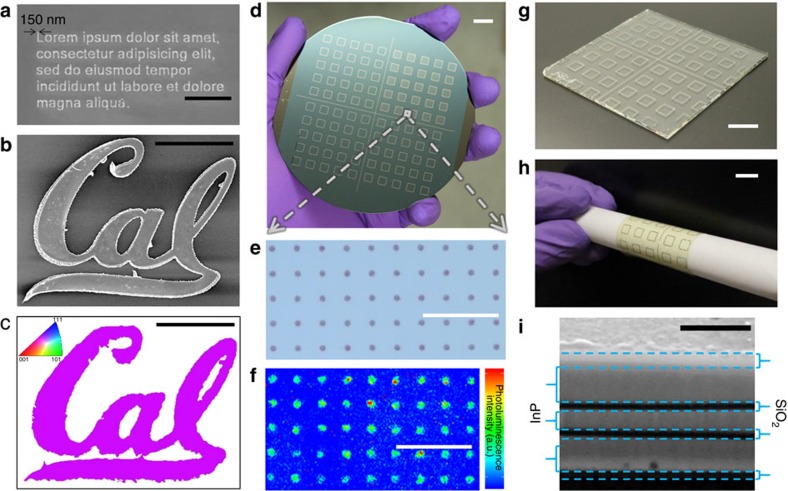
Scalability and growth on ‘novel' substrates. (**a**) SEM image of InP text with a linewidth of 150 nm. Scale bar, 3 μm. (**b**) An SEM image and corresponding (**c**) EBSD map of a single-crystalline UC Berkeley ‘*Cal*' logo grown via TLP crystal growth. Scale bar, 30 μm. The Cal script logo is a federally registered trademark and may not be used without permission of The Regents of the University of California. (**d**) An optical image of a 4-inch Si/SiO_2_ wafer patterned with arrays of 3 μm diameter InP dots located within each square InP box. Scale bar, 1 cm. (**e**) Zoomed in optical image of an array of dots on the wafer and (**f**) their corresponding photoluminescence image. Scale bar, 50 μm. (**g**) An optical image of patterned InP dots grown on a ∼6 × 6 cm borosilicate glass slide. Scale bar, 1 cm. (**h**) An optical image of the patterned InP dot array transferred onto a PET substrate wrapped around a glass tube with a diameter of 1.5 cm. Scale bar, 1 cm. (**i**) Cross-sectional SEM image of three layers of InP grown with 30 nm of SiO_x_ between each layer. Scale bar, 500 nm.

To demonstrate the versatility of the TLP crystal growth technique, the world's smallest version of the ‘Lorem Ipsum' placeholder text is written in crystalline InP lettering with a stroke width of 150 nm (Fig. 2a). In addition, a single-crystalline Berkeley ‘Cal' logo with dimensions of 80 × 60 μm is shown in Fig. 2b with its structural, compositional and optoelectronic properties characterized via EBSD, EDS mapping and photoluminescence imaging (Fig. 2c, Supplementary Fig. 7), respectively. While letters are used for demonstration purposes, the geometric degrees of freedom available to grow single-crystalline materials with TLP crystal growth are of great significance for the fabrication of practical electronic and photonic devices.

One major advantage of TLP crystal growth is ease of scalability. Unlike traditional III–V growth where both group III and V elements are introduced in the vapour phase, only the group V element is in the vapour phase, and the geometry is fixed by the template. This allows for simple reactor designs which can be easily scaled up to large scales. Furthermore, as growth only occurs when both group III and group V elements are present, the templates essentially provide a self-limiting growth mechanism, preventing unwanted thickness or compositional variation across a wafer. As a demonstration, Fig. 2d–f shows optical images as well as a corresponding photoluminescence image of arrays of InP circles outlined by InP bars, grown across a full 4-inch Si/SiO_2_ wafer using a simple cold-wall furnace.

The relatively low temperature required for TLP crystal growth also allows for a broad range of substrates upon which single-crystalline III–V's can be grown. As an example, InP circles were directly written onto a borosilicate glass slide (Fig. 2g) and their optical quality was verified via photoluminescence imaging (Supplementary Fig. 8). In addition, the grown InP can be fully transferred onto plastic substrates for applications where a flexible substrate is desired. As an example, Fig. 2h and Supplementary Fig. 9, show an optical image and the corresponding photoluminescence image, respectively, of the InP circle arrays transferred onto a polyethylene terephthalate (PET) substrate using polyamic acid^26^.

A unique feature of the TLP crystal growth process is that complex 3D architectures can be achieved, beyond the limits of traditional processes. For instance, multilayers of InP single crystals separated by amorphous SiO_x_ layers can be grown in one cycle by starting with substrates consisting of multilayer In/SiO_x_ layers, as demonstrated in the cross-sectional SEM image in Fig. 2i. This can have broad implications for future design of monolithic 3D electronics.

### *In situ* doping

**Figure 3:**
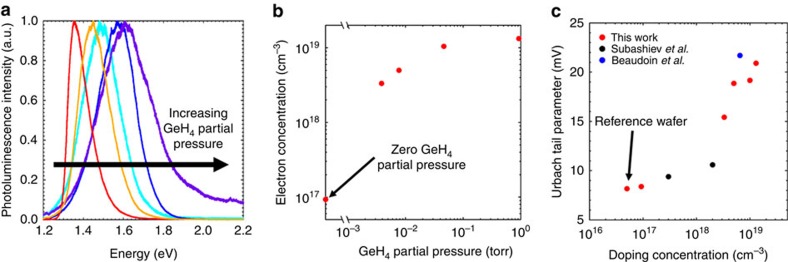
*In situ* doping. (**a**) Photoluminescence spectra of five InP samples grown with GeH_4_ pressures of 0, 3.9, 7.7, 46 and 900 mTorr of GeH_4_. As the GeH_4_ partial pressure is increased, the peak of the photoluminescence spectra blue-shifts, signifying higher electron concentrations. (**b**) A plot of the electron concentration, approximated from the peak position of the photoluminescence spectra using the Burstein–Moss effect, as a function of the GeH_4_ partial pressure. (**c**) The Urbach tail parameter is plotted versus the approximated doping level showing that the crystal quality is on par with that of single-crystalline wafers/films at different levels of doping.

A critical component of semiconductor growth is the ability to tune the optical and electrical properties; in particular, the doping concentration. To explore this capability, GeH_4_ gas was introduced into the growth chamber during growth to achieve controlled *in situ * n-type Ge doping of InP. Figure 3a shows the normalized photoluminescence spectra of InP single crystals grown under different partial pressures of GeH_4_. Significant blue-shifting of the photoluminescence peak versus a lightly doped reference wafer (Supplementary Fig. 10) is observed as the GeH_4_ partial pressure is increased owing to the Burstein–Moss effect, indicating increased electron concentrations^27^,^28^,^29^. The electron concentrations, approximated from the photoluminescence peak energies (see Methods section), are plotted versus the GeH_4_ partial pressure in Fig. 3b indicating that TLP grown InP can be doped up to a degenerate level of ∼1.3 × 10^19^ cm^−3^, near the upper limit of electron doping in InP^30^. From the photoluminescence spectra, the Urbach tail parameter is also extracted and plotted versus the carrier concentration level in Fig. 3c (ref. 31). The Urbach tail parameter is an important figure of merit regarding the band edge sharpness arising from crystal defects, thermal vibrations and charged impurities^32^,^33^. As can be seen, the Urbach tails of our non-epitaxial TLP grown samples are similar to the values reported in literature for InP single crystal wafers at various respective levels of doping^34^,^35^.

### Electronic characterization

**Figure 4:**
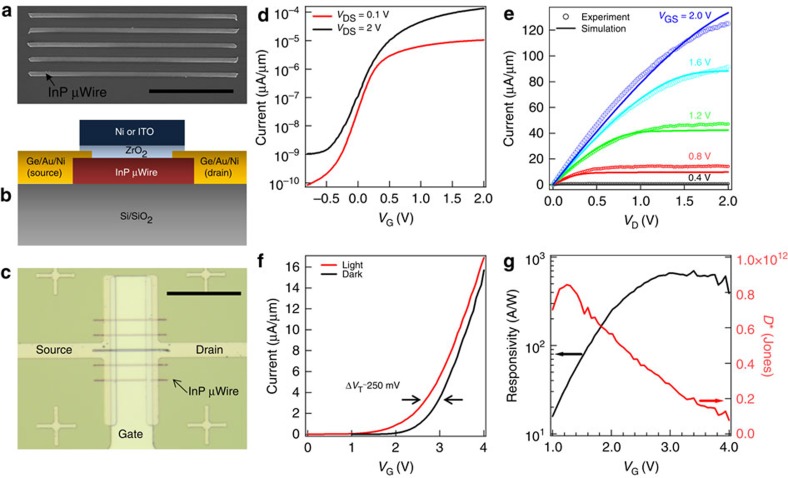
Electronic characterization. (**a**) An SEM image of typical 1-μm wide InP μWires used for MOSFET fabrication. Scale bar, 20 μm. (**b**) A cross-sectional schematic of the InP MOSFETs along with (**c**) an optical image of a MOSFET with 5 μwires as the channel. Scale bar, 50 μm. (**d**) The transfer *I*_DS_-*V*_GS_ and (**e**) output *I*_DS_-*V*_DS_ curves of an InP transistor with a single μWire as the channel and a 3 μm gate length. (**f**) The transfer characteristics of a photo-MOSFET in dark and exposed to 15.6 mW cm^−2^ of light, showing a large threshold voltage shift of ∼250 mV. (**g**) The responsivity and detectivity of the photo-MOSFET are plotted versus the gate voltage bias.

The electronic quality and practical utility of TLP-growth InP in the shape of microwires (μWires) were explored by fabricating long-channel Schottky n-type metal-oxide-semiconductor field effect transistors (MOSFETs) with top gates (Fig. 4a–c). The transfer and output characteristics of an InP transistor on a Si/SiO_2_ substrate with a gate length of 3 μm and body thickness of ∼125 nm are shown in Fig. 4d,e, respectively. The device exhibits an ON-current of 120 μA μm^−1^ at *V*_GS_=*V*_DS_=2 V with an ON/OFF current ratio of >10^5^ and peak extrinsic transconductance of 100 S μm^−1^, which is excellent for a long-channel device. An effective electron mobility of μ=675 cm^2^ V^−1^ s^−1^ is extracted from device simulations (see Methods section), which compares favourably with unpassivated InP nanowire/microwire MOSFETs in literature^9^, illustrating the excellent electronic quality of the InP grown here. In addition, top-gated InP photo-MOSFETs were also fabricated on Si/SiO_2_ substrates using the device structure above except that a transparent indium tin oxide (ITO) gate electrode is used with a channel length of *L*_G_=20 μm. The device electrical characteristics were measured under dark and under steady-state illumination from a bandpass filtered white light source with an optical intensity of 15.6 mW cm^−2^. The device exhibits a strong photoresponse (Fig. 4f) with a peak responsivity of ∼700 A W^−1^ at *V*_GS_=3.4 V (Fig. 4g). Furthermore, the specific detectivity (D*) of this device displays a maximum of ∼8.4 × 10^11^ Jones at room temperature, comparable to state-of-the-art single-crystalline epitaxial InGaAs detectors^14^.

## Discussion

In conclusion, we have demonstrated a technique that enables direct ‘writing' of optoelectronic-quality single-crystalline III–V semiconductors on amorphous substrates. The elimination of the requirement for lattice-matched substrates as well as the improved scalability of this growth mode enables ubiquitous integration of III–V semiconductors for a wide range of applications on user-defined substrates. While InP was used as a model growth system in this work, the TLP crystal growth method is one that, from a thermodynamic and kinetic point of view, is expected to be applicable to other technologically important III–V's. As an example, proof of concept demonstrations using the TLP process to grow GaP and InSb are shown in Supplementary Fig. 11. In addition, single crystals grown via the TLP growth process may potentially be utilized as a virtual substrate in epitaxial growth processes, allowing for the realization of high-quality semiconductor heterostructures grown directly onto amorphous substrates. Future work on control of crystal orientation of individual patterns may further extend the tunability of the TLP growth mode, for instance, by making nucleation of a specific orientation thermodynamically favourable through surface engineering of the nucleation layer or via the introduction of geometric constraints using principles from graphoepitaxy^12^.

## Methods

### Patterning and growth of InP

First, a clean Si wafer with a 50-nm thick thermal oxide was lithographically patterned with the desired InP shape (Supplementary Fig. 1a). For the glass sample in Fig. 2g, a borosilicate glass slide was patterned instead. A thin 1–10 nm thick MoO_x_ layer was evaporated (Supplementary Fig. 1b) followed by evaporation of In of the desired thickness and a 10–100-nm thick SiO_x_ layer (Supplementary Fig. 1c). As SiO_2_ has a high surface energy for nucleation^24^, the MoO_x_ layer helps to promote nucleation of the InP^25^. To obtain a smooth In film, the evaporation of the In and SiO_x_ bilayer was done with the substrate chuck cooled to <150 K using liquid N_2_. The whole MoO_x_/In/SiO_x_ stack was then lifted off (Supplementary Fig. 1d). After liftoff, angled evaporation was utilized to coat the exposed side regions of the In with SiO_x_ with thicknesses ranging from 4 to 50 nm (Supplementary Fig. 1e,f). During the growth, the SiO_x_ template confines the liquid In so that the resulting InP crystal has the same shape as that of the original In pattern. Growth of the InP patterns for EBSD crystal analysis and transistors were carried out in a hot-wall CVD tube furnace. In all, 10% PH_3_ in H_2_ was used as the phosphorous source and was further diluted to the desired dilution. Growth of the 4-inch wafer, sample on glass and doping-dependent studies were done in a cold-wall CVD system. In all, 10% GeH_4_ in H_2_ was used as the Ge dopant source. The samples were grown for 10–20 min at pressures of 100–300 Torr (partial PH_3_ pressure of 0.1–10 Torr) and growth temperatures ranging between 500 and 535 °C.

### EBSD characterization

EBSD characterization was carried out in an FEI Quanta SEM with an Oxford Instruments EBSD detector. Analysis of the maps were done by the Oxford Aztec and Tango software programs. Orientation maps were generated and plotted using the inverse pole figure colour scheme. Twin boundary removal was done by ignoring the <111> 60° rotational boundaries within the crystals and plotting the surface orientation of each grain.

### Photoluminescence spectra and imaging

Photoluminescence spectra were taken by a HORIBA LabRAM HR800 tool with a 532 nm excitation wavelength. For photoluminescence imaging, a red LED was used as the excitation light source and images were taken by an Andor silicon CCD camera through an optical microscope with a GaAs wafer used to filter out the irradiation wavelengths.

### Electron concentration extraction

The electron concentration, *n*, can be approximated using the equation^27^:


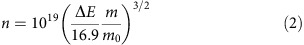


where *ΔE* is the shift of the photoluminescence peak energy from an undoped reference (1.34 eV taken from a 5 × 10^16^ cm^−3^ doped reference wafer, Supplementary Fig. 10) and *m/m*_*0*_ is the ratio of the effective electron mass of InP to the free electron mass.

### Urbach tail fitting

The absorption at the band edge, is related to the photoluminescence spectra by the van Roosbroeck–Schockley equation by^27^


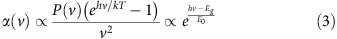


where *P(v)* is the photoluminescence intensity as a function of frequency *v*, h is Planck's constant, and k*T* is the thermal energy (25.6 mV at room temperature). *E*_0_, the slope at the absorption band edge, is the Urbach tail parameter, which describes the sharpness at the band edge and is a good indicator of crystal and optoelectronic quality.

### Device fabrication

InP microwires with dimensions of 1 × 50μm and thickness of 125 nm were grown using TLP crystal growth as described above. Photolithography was used to lithographically define the source/drain contacts followed by evaporation of 3/10/40 nm of Ge/Au/Ni and liftoff. The source/drain contacts were subsequently annealed at 375 °C for 5 min to alloy the Ge with InP in the contact regions to improve contact resistance. In all, 10 nm of ZrO_2_ was then deposited via atomic layer deposition at a temperature of 200 °C. Finally, photolithography was used once again to define the gate electrode. For the MOSFETs, 40 nm of Ni was evaporated as the top gate metal while for the photo-MOSFETs, 30 nm of ITO was deposited via sputtering instead to allow optical access to the channel.

### Sentaurus simulations

Detailed semi-classical drift-diffusion simulations were carried out utilizing the Sentaurus Device simulator to accurately model the device performance. The parameter extraction was carried out by first matching the subthreshold region (−0.2 V<*V*_GS_<0.15 V) utilizing InP/ZrO_2_ surface interface traps and gate work function as the fitting parameters and performing a least squares error fit, enabling accurate simulation of the mobile charge versus gate voltage. The mobility and series resistance of the device were extracted by minimizing the least squares error for all the *I*_DS_-*V*_DS_ curves (0 V<*V*_DS_<2 V) for *V*_GS_=0.4, 0.8, 1.2, 1.6 and 2 V simultaneously.

## Additional information

**How to cite this article:** Chen, K. *et al.* Direct growth of single-crystalline III–V semiconductors on amorphous substrates. *Nat. Commun.* 7:10502 doi: 10.1038/ncomms10502 (2016).

## Supplementary Material

Supplementary Information Supplementary Figures 1-11, Supplementary Note 1 and Supplementary References.

## References

[b1] Kroemer H. Heterostructure bipolar transistors and integrated circuits. Proc. IEEE 70, 13–25 (1982).

[b2] Ko H. *et al.* Ultrathin compound semiconductor on insulator layers for high-performance nanoscale transistors. Nature 468, 286–289 (2010). 21068839 10.1038/nature09541

[b3] del Alamo J. A. Nanometre-scale electronics with III–V compound semiconductors. Nature 479, 317–323 (2011). 22094691 10.1038/nature10677

[b4] Yoon J. *et al.* GaAs photovoltaics and optoelectronics using releasable multilayer epitaxial assemblies. Nature 465, 329–333 (2010). 20485431 10.1038/nature09054

[b5] Wallentin J. *et al.* InP nanowire array solar cells achieving 13.8% efficiency by exceeding the ray optics limit. Science 339, 1057–1060 (2013). 23328392 10.1126/science.1230969

[b6] Nakamura S., Mukai T. & Senoh M. Candela-class high-brightness InGaN/AlGaN double-heterostructure blue-light-emitting diodes. Appl. Phys. Lett. 64, 1687–1689 (1994).

[b7] Campbell J. C. Recent advances in telecommunications avalanche photodiodes. J. Light Technol. 25, 109–121 (2007).

[b8] Cho A. Y. & Arthur J. R. Molecular beam epitaxy. Prog. Solid State Chem. 10, 157–191 (1975).

[b9] Dapkus P. D., Manasevit H. M., Hess K. L., Low T. S. & Stillman G. E. High purity GaAs prepared from trimethylgallium and arsine. J. Cryst. Growth 55, 10–23 (1981).

[b10] Dijkkamp D. *et al.* Preparation of Y-Ba-Cu oxide superconductor thin films using pulsed laser evaporation from high Tc bulk material. Appl. Phys. Lett. 51, 619–621 (1987).

[b11] Matthews J. W. & Blakeslee A. E. Defects in epitaxial multilayers: I. Misfit dislocations. J. Cryst. Growth 27, 118–125 (1974).

[b12] Ohring M. Materials Science of Thin Films Academic Press (2001).

[b13] Duan X., Huang Y., Cui Y., Wang J. & Lieber C. M. Indium phosphide nanowires as building blocks for nanoscale electronic and optoelectronic devices. Nature 409, 66–69 (2001). 11343112 10.1038/35051047

[b14] Morales A. M. & Lieber C. M. A laser ablation method for the synthesis of crystalline semiconductor nanowires. Science 279, 208–211 (1998). 9422689 10.1126/science.279.5348.208

[b15] Wagner R. S. & Ellis W. C. Vapor-liquid-solid mechanism of single crystal growth. Appl. Phys. Lett. 4, 89–90 (1964).

[b16] Chung S.-W., Yu J.-Y. & Heath J. R. Silicon nanowire devices. Appl. Phys. Lett. 76, 2068–2070 (2000).

[b17] Björk M. T. *et al.* One-dimensional heterostructures in semiconductor nanowhiskers. Appl. Phys. Lett. 80, 1058–1060 (2002).

[b18] Pan Z. W., Dai Z. R. & Wang Z. L. Nanobelts of semiconducting oxides. Science 291, 1947–1949 (2001). 11239151 10.1126/science.1058120

[b19] Wang Z. L. Zinc oxide nanostructures: growth, properties and applications. J. Phys. Condens. Matter 16, R829 (2004).

[b20] Heurlin M. *et al.* Continuous gas-phase synthesis of nanowires with tunable properties. Nature 492, 90–94 (2012). 23201685 10.1038/nature11652

[b21] Choi J. H. *et al.* Nearly single-crystalline GaN light-emitting diodes on amorphous glass substrates. Nat. Photon. 5, 763–769 (2011).

[b22] Shon J. W., Ohta J., Ueno K., Kobayashi A. & Fujioka H. Fabrication of full-color InGaN-based light-emitting diodes on amorphous substrates by pulsed sputtering. Sci. Rep. 4, 5325 (2014). 24954609 10.1038/srep05325 PMC4066259

[b23] Joyce H. J. *et al.* Electronic properties of GaAs, InAs and InP nanowires studied by terahertz spectroscopy. Nanotechnology 24, 214006 (2013). 23619012 10.1088/0957-4484/24/21/214006

[b24] Kapadia R. *et al.* A direct thin-film path towards low-cost large-area III–V photovoltaics. Sci. Rep. 3, 2275 (2013). 23881474 10.1038/srep02275 PMC3721076

[b25] Kapadia R. *et al.* Deterministic nucleation of InP on metal foils with the thin-film vapor–liquid–solid growth mode. Chem. Mater. 26, 1340–1344 (2014).

[b26] Cao Q. *et al.* Medium-scale carbon nanotube thin-film integrated circuits on flexible plastic substrates. Nature 454, 495–500 (2008). 18650920 10.1038/nature07110

[b27] Wallentin J. *et al.* Probing the wurtzite conduction band structure using state filling in highly doped InP nanowires. Nano Lett. 11, 2286–2290 (2011). 21604708 10.1021/nl200492g

[b28] Bennett B. R., Soref R. A. & del Alamo J. A. Carrier-induced change in refractive index of InP, GaAs and InGaAsP. IEEE J. Quantum Electron. 26, 113–122 (1990).

[b29] Liu C., Dai L., You L. P., Xu W. J. & Qin G. G. Blueshift of electroluminescence from single n-InP nanowire/p-Si heterojunctions due to the Burstein–Moss effect. Nanotechnology 19, 465203 (2008). 21836237 10.1088/0957-4484/19/46/465203

[b30] Yu K. M., Moll A. J. & Walukiewicz W. Amphoteric behavior and precipitation of Ge dopants in InP. J. Appl. Phys. 80, 4907–4915 (1996).

[b31] Basu P. K. Theory of Optical Processes in Semiconductors : Bulk and Microstructures Clarendon Press (1997).

[b32] Iribarren A., Castro-Rodríguez R., Sosa V. & Peña J. L. Modeling of the disorder contribution to the band-tail parameter in semiconductor materials. Phys. Rev. B 60, 4758–4762 (1999).

[b33] Iribarren A., Castro-Rodríguez R., Sosa V. & Peña J. L. Band-tail parameter modeling in semiconductor materials. Phys. Rev. B 58, 1907–1911 (1998).

[b34] Subashiev A. V., Semyonov O., Chen Z. & Luryi S. Urbach tail studies by luminescence filtering in moderately doped bulk InP. Appl. Phys. Lett. 97, 181914 (2010).

[b35] Beaudoin M., Johnson S. r., Devries A. j. g., Mohades-Kassai A. & Tiedje T. Temperature dependence of the optical absorption edge in indium phosphide. MRS Proc. 421,, 367–372 (1996).

