# Supplementary material for: Direct growth of single-crystalline III–V semiconductors on amorphous substrates
Source: Nat Commun. 2016 Jan 27;7:10502. doi: 10.1038/ncomms10502 (PMC4737854; doi:10.1038/ncomms10502)
Supplement: Supplementary Information — Supplementary Figures 1-11, Supplementary Note 1 and Supplementary References. [file ncomms10502-s1.pdf]

Supplementary Figures

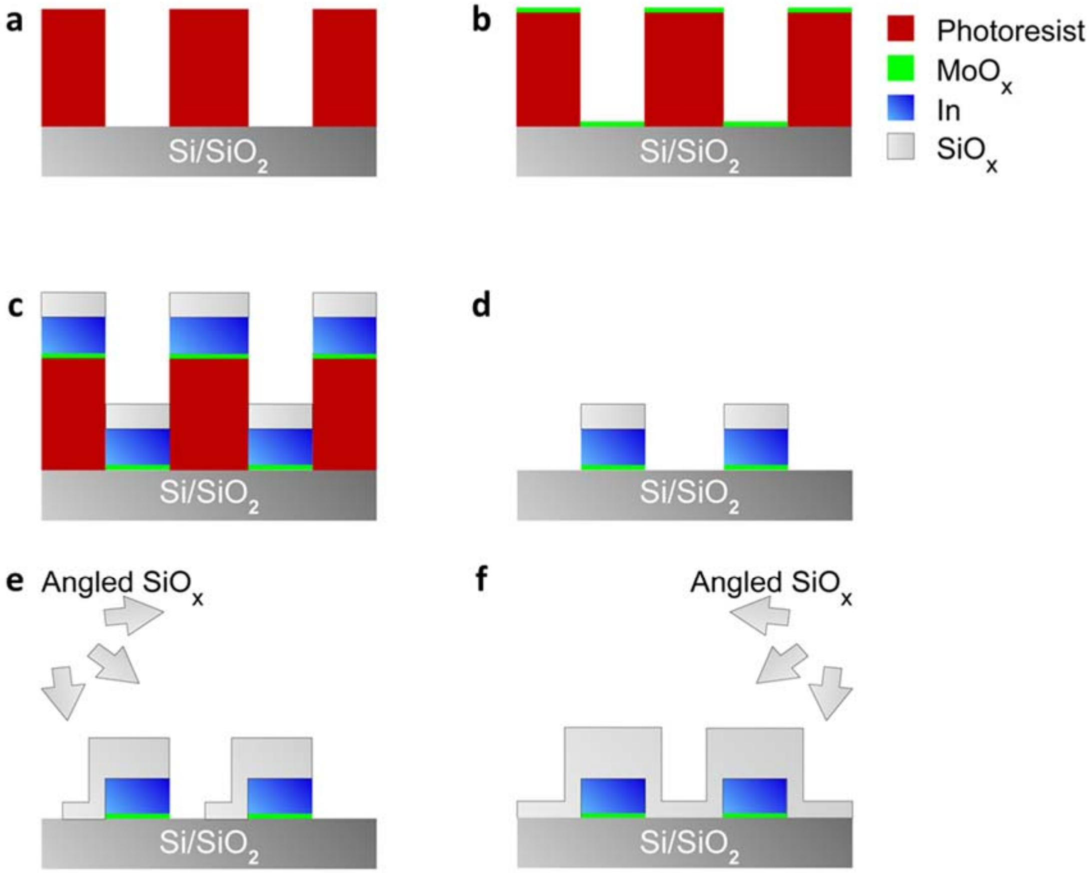

Supplementary Figure 1: Sample preparation for TLP crystal growth. (a) The sample is first patterned via photolithography and (b) a thin 1-10 nm thick MoO_x layer is evaporated. (c) Then, a bilayer of In and SiO_x is evaporated while the substrate chuck is cooled below 150° C using liquid N_2. (d) After evaporation, the whole stack is lifted off and (e, f) SiO_x is evaporated from 2-3 angles in order to fully encapsulate the In.

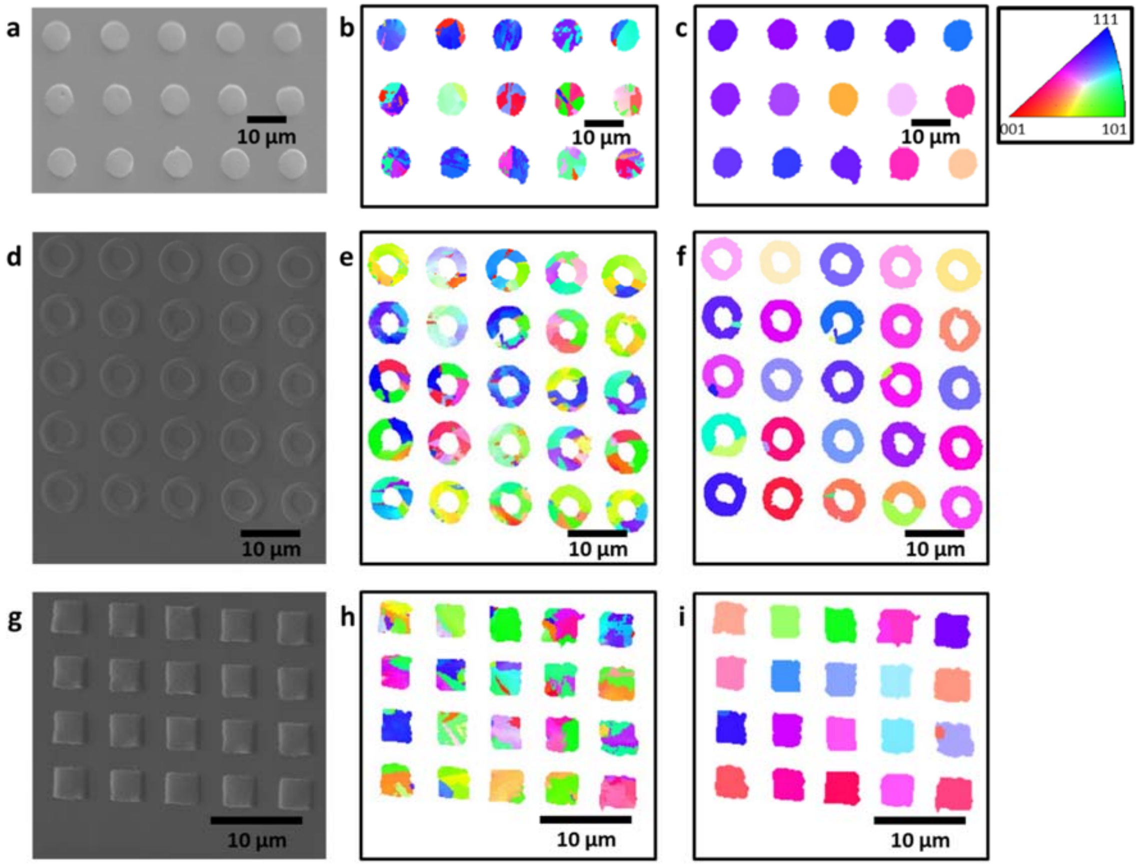

Supplementary Figure 2: EBSD map of various grown shapes. (a) SEM image of patterned InP circles along with corresponding EBSD maps (b) before and (c) after removal of the <111> 60° twin boundaries. (d) SEM image of patterned InP rings along with corresponding EBSD maps (e) before and (f) after removal of the <111> 60° twin boundaries. It can be seen that some of the rings consist of two grains. (g) SEM image of patterned InP squares along with corresponding EBSD maps (h) before and (i) after removal of the <111> 60° twin boundaries. One of the squares consists of two grains, while the rest are all single crystals. The samples were grown at a partial PH_3 pressure of 10 Torr.

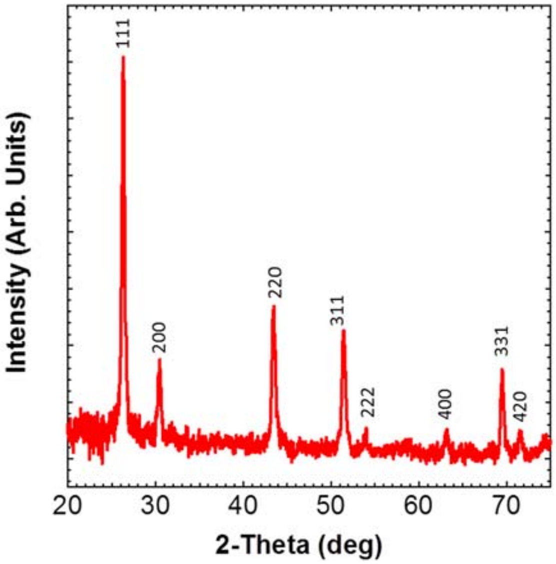

Supplementary Figure 3: X-ray diffraction characterization. XRD diffractogram of an array of InP circles displaying only peaks from the zincblende phase, indicating complete conversion of In into InP, within the resolution limit of XRD.

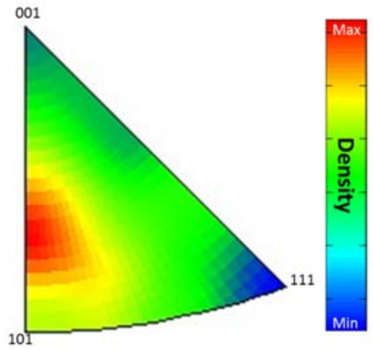

Supplementary Figure 4: Orientation Distribution Inverse Pole Map. The orientation distribution in the normal direction of an array of InP circles obtained from EBSD indicate a slight preferential orientation in the [1 0 n] direction, with n ranging between 1 and 2.

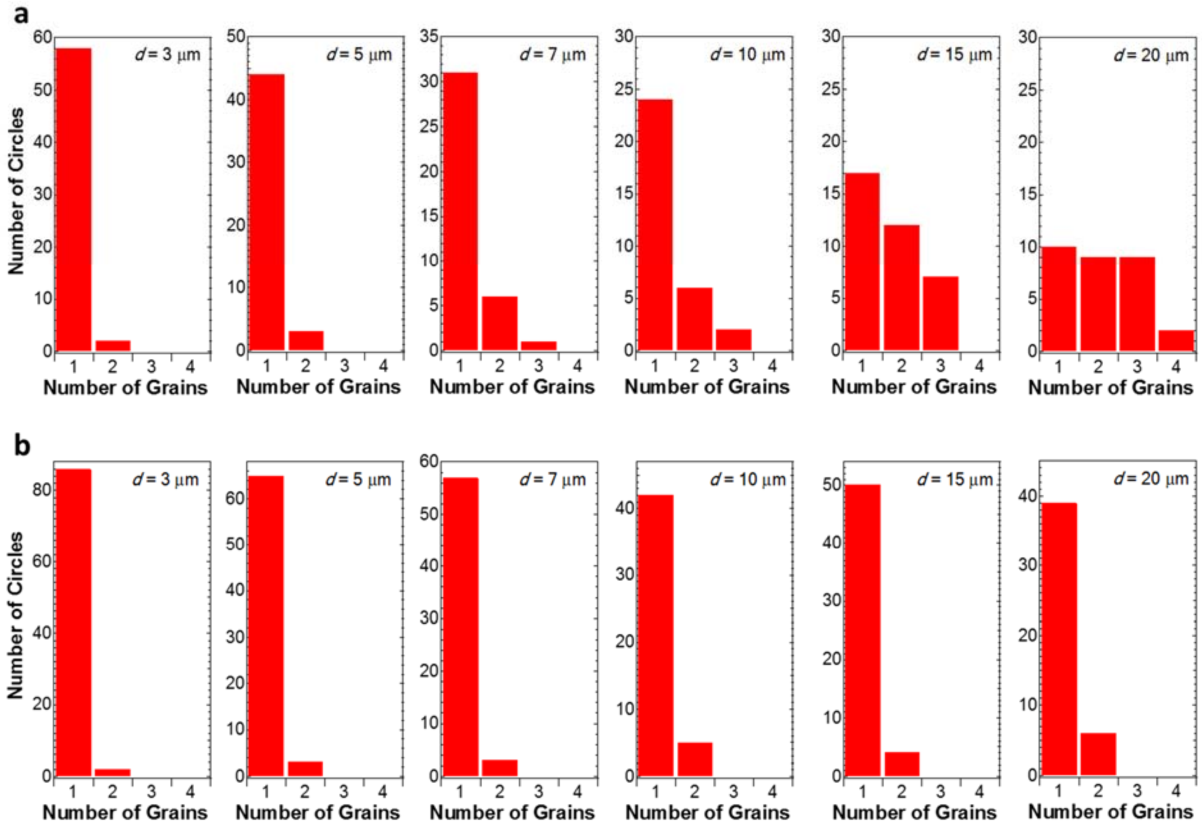

Supplementary Figure 5: Histograms of Circle Grains. (a,b) The statistical distribution of the number of circles vs. number of grains plotted as histograms for PH_3 partial pressures of (a) 1 Torr and (b) 0.1 Torr for circles of diameter 3 μm, 5 μm, 7 μm, 10 μm, 15 μm, and 20 μm.

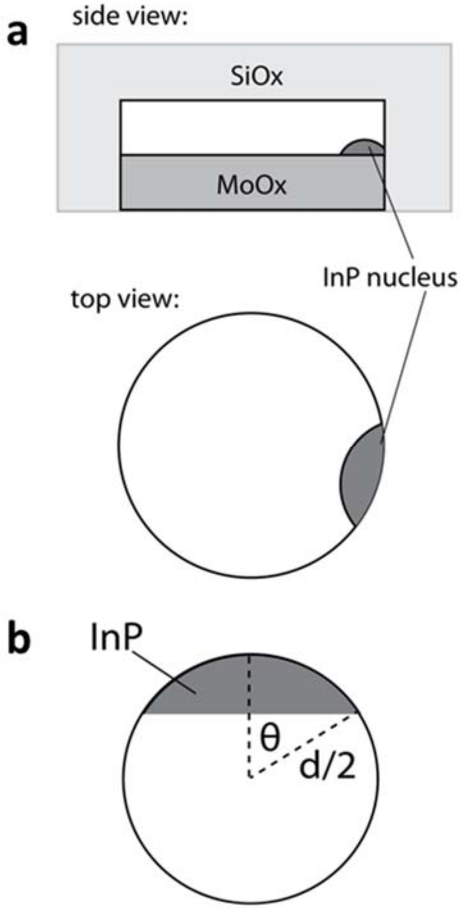

Supplementary Figure 6: Nucleation model (a) Nucleation along the edges of the circles. (b) The geometry assumed in computing the results in Eqn. (6) of Supplementary Note2. The growing nucleus is assumed to block nucleation sites as it advances. This, in turn, will reduce the net nucleation rate within the circle (all other factors being equal). Equation (6) of Supplementary Note 2 is derived by noting that the total area of the nucleus increases approximately linearly with time, and this enables one to compute $\theta(t)$, and thereby the edge length available for nucleation of additional grains. d is the diameter of the circle.

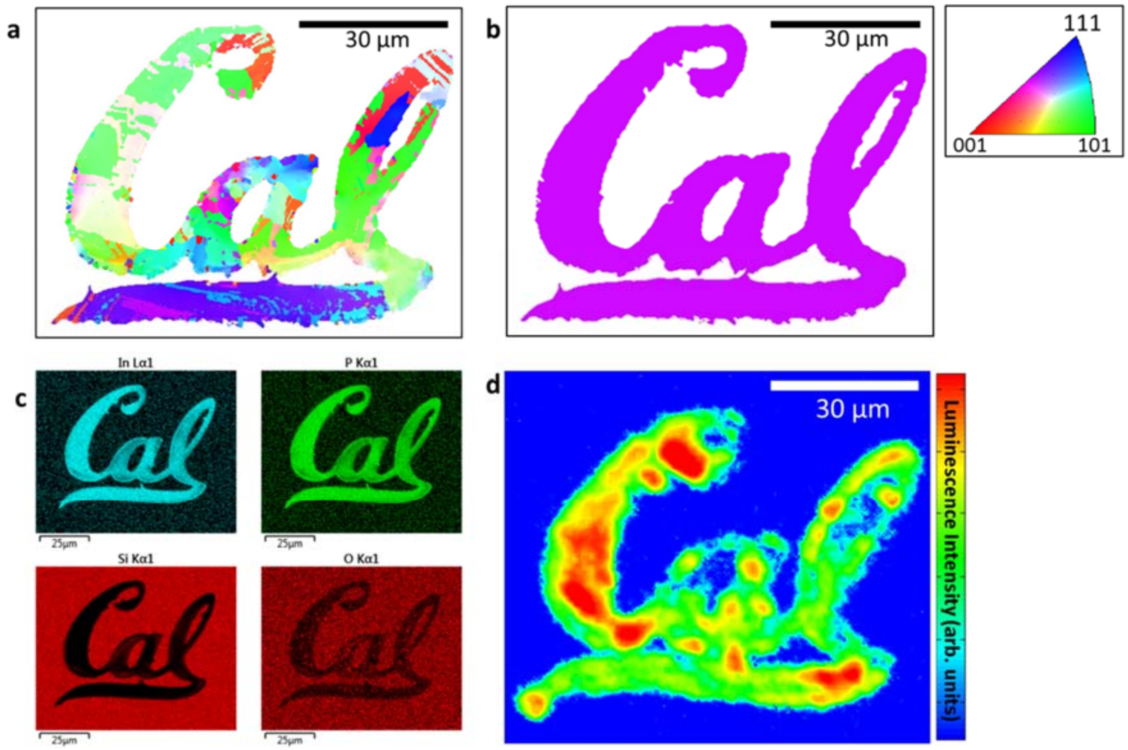

Supplementary Figure 7: Characterization of Single Crystal "*Cal*" (a) EBSD map of the "*Cal*" shown in Figure 2b before and (b) after twin boundary removal. (c) The EDS elemental map of the region containing the single crystal "*Cal*" for In, P, O, and Si. The intensity of each color (versus black background) indicates the amount of that element mapped within the region. The extracted InP stoichiometry shows an In to P ratio of 1:1. (d) The PL image of the single crystalline InP "*Cal*" showing that it is optically active.

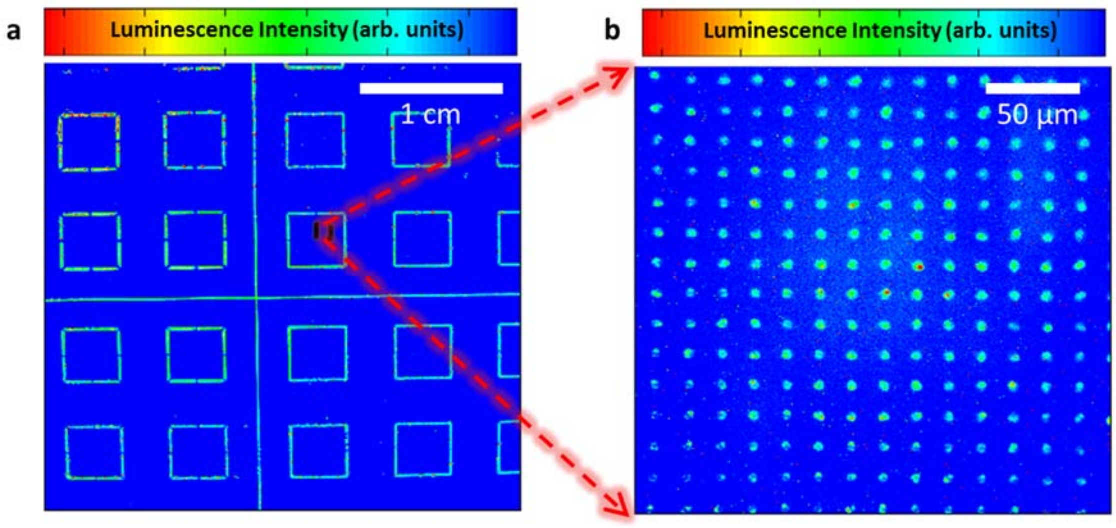

Supplementary Figure 8: PL imaging of patterned InP grown on borosilicate glass. (a) The PL image of a ~4x4 cm patterned area of 3 µm InP circles within large InP square frames grown on borosilicate glass along with (b) a zoomed in PL intensity map of the actual circle arrays.

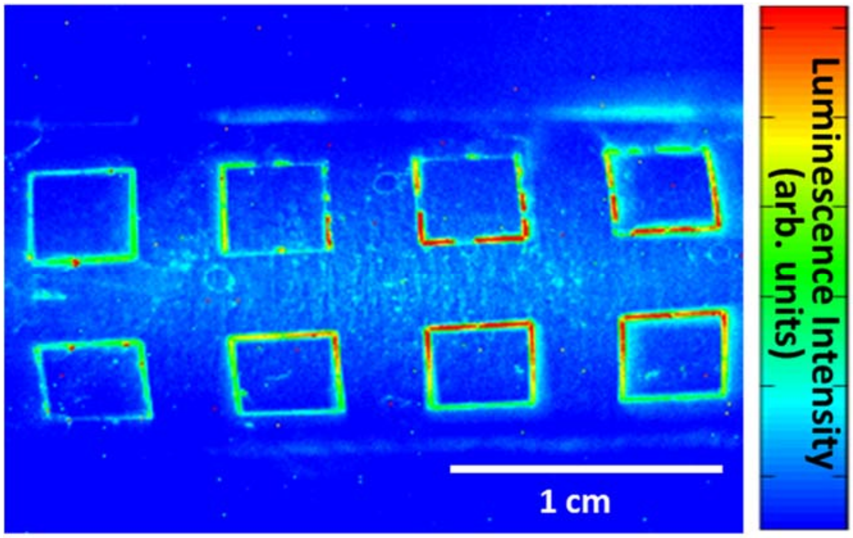

Supplementary Figure 9: PL imaging of InP circle arrays transferred onto plastic. As the polyimide used in the transfer process does slightly fluoresce in the infrared regime, a weak outline of the test tube upon which the plastic substrate is wrapped around is also seen in the PL image.

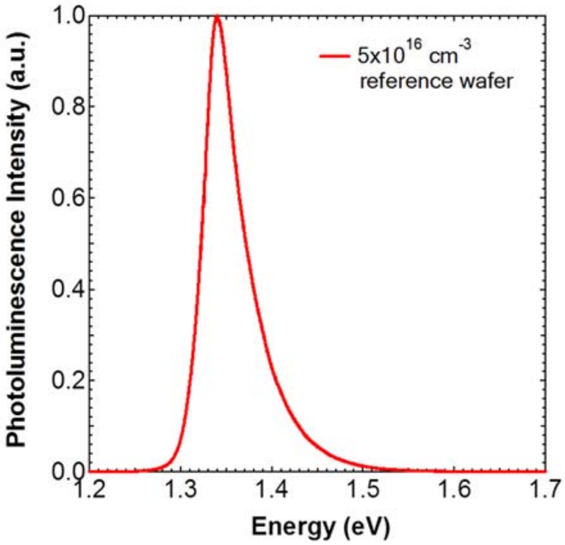

Supplementary Figure 10: PL spectra of an InP reference wafer. PL spectra of a single crystal InP reference wafer with a doping level of 5×10^{16} cm^{-3}

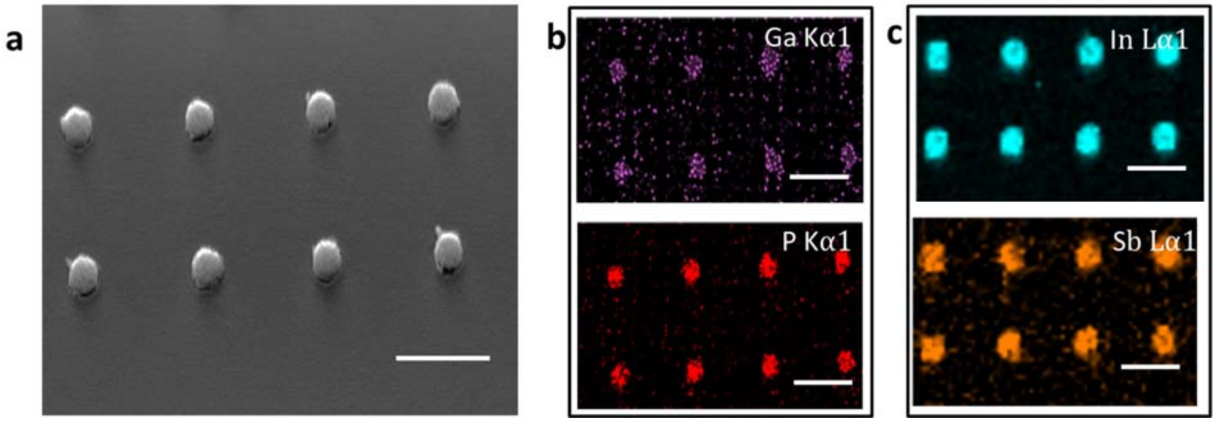

Supplementary Figure 11: TLP Growth of GaP and InSb. a) SEM image of patterned GaP circles and b) the corresponding EDS maps of the region for Ga and P. c) EDS maps of In and Sb of a similarly patterned region of InSb. Scale bars for all images are 5 µm.

Supplementary Notes

Supplementary Note 1: Twinning within InP.

From EBSD measurements results shown in Supplementary Figure 2 and Supplementary Figure 7, it can be seen that the crystals have <111>60° rotational twin boundaries, which is commonly seen in the growth of III-V materials[1] and in particular InP due to its low stacking fault energy. Previously, nanowires grown via the vapor-liquid-solid (VLS) growth mode often exhibit stacking fault[2–4] due to this reason. Upon using the Tango analysis software to disregard twin boundaries in the EBSD analysis, it is revealed that the individual features grown in this work do show up as single crystals, indicating that all features do indeed grow out from a single nucleus.

Despite the existence of the twin boundaries, it can be seen from Urbach tail measurements, as shown in Figure 3c, that the Urbach tail parameter is on par with a single crystalline reference wafer, as well as reported Urbach tail parameters in literature for single crystalline InP[5,6]. In addition, the electronic and optoelectronic characterization in Figure 4 of devices made from the single crystals grown via the TLP crystal growth method are still of extremely high quality, on par with commercial single crystalline InGaAs photodetectors[7].

Supplementary Note 2: Nucleation model for number of grains per circle vs. size

Consider the nucleation of the InP solid phase within In liquid supersaturated with P. The nucleation process is a stochastic one, in which the nucleation rate, Γ, is time dependent, $\Gamma = \Gamma(t)$. Within this model, the probability that k grains nucleate between the time $t = 0$ and $t = \tau$ is given by:

$$P(k,\tau) = \frac{e^{-N(\tau)}}{k!}N(\tau)^k, \text{with } N(\tau) = \int_0^\tau dt\Gamma(t) \tag{1}$$

We define the time $t = 0$ to be that time at which the first grain nucleates in the circle and t_{growth} to be the time at which the disk is completely transformed.

The distribution governing the number of grains nucleated after the first is given by $P(k, t_{\mathrm{growth}})$, and the final average number of grains within the circle, N_{grains}, is given by:

$$\begin{aligned} N_{\mathrm{grains}} &= 1 + \langle k \rangle \\ &= 1 + N(t_{\mathrm{growth}}) \end{aligned} \tag{2}$$

Consider now the case that nucleation takes place at a constant rate per available nucleation site in the "corners" of the circles (See Supplementary Fig. 6). Under these circumstances, the nucleation rate can be written as

$$\Gamma(t) = \alpha(t)\gamma_0\pi d \tag{3}$$

where $\alpha(t)$, a dimensionless quantity, is the fraction of the edge length available for nucleation at time t, πd is the total edge length available for nucleation at time $t = 0$, and γ_0 is the average nucleation rate per unit of edge length throughout the growth process.

Further progress can be made if one assumes a growth geometry for the initial nucleus, and that the time to form the first nucleus is small in comparison to the total growth time.

In the experiments, the SiO_x cap on the top of the In circles is designed to be much thicker than that on the sides (Supplementary Figure 1). As such, it can be assumed that the P is only entering through the sides of the In circles, so that the number of P atoms entering the liquid, per unit time, $\dot{n}_{\mathrm{P}}$ is given by:

$$\dot{n}_{\mathrm{P}} = J_{\mathrm{P}}\pi h d \tag{4}$$

where h is the original thickness of the In circle, d the original diameter of the circle, and J_{P} the average flux rate of the P through the SiO_x during the growth. Given this average flux rate, the time for the first nucleus to completely transform the circle is:

$$t_{\mathrm{growth}} \approx \frac{d}{4J_{\mathrm{P}}\Omega_{\mathrm{In}}}\,, \qquad (5)$$

Using the geometry shown in Supplementary Figure 6, one finds:

$$\begin{aligned} \langle k \rangle &= N\left(t_{\mathrm{growth}}\right) \\ &= \frac{\pi}{8}\frac{\gamma_0}{J_{\mathrm{P}}\Omega_{\mathrm{In}}} d^2, and \\ N_{\mathrm{grains}} &= 1 + \frac{\pi}{8}\frac{\gamma_0}{J_{\mathrm{P}}\Omega_{\mathrm{In}}} \end{aligned} \qquad (6)$$

So the average number of grains in the circles should increase with diameter according to $N_{\mathrm{grains}} = 1 + \beta d^2$, with β depending on the imposed growth conditions.

Supplementary References:

1. Hurle, D. T. J. & Rudolph, P. A brief history of defect formation, segregation, faceting, and twinning in melt-grown semiconductors. *J. Cryst. Growth* **264,** 550–564 (2004).
2. Algra, R. E. *et al.* Twinning superlattices in indium phosphide nanowires. *Nature* **456,** 369–372 (2008).
3. Johansson, J. *et al.* Structural properties of <111>B -oriented III–V nanowires. *Nat. Mater.* **5,** 574–580 (2006).
4. Korgel, B. A. Semiconductor nanowires: Twins cause kinks. *Nat. Mater.* **5,** 521–522 (2006).
5. Subashiev, A. V., Semyonov, O., Chen, Z. & Luryi, S. Urbach tail studies by luminescence filtering in moderately doped bulk InP. *Appl. Phys. Lett.* **97,** 181914 (2010).
6. Beaudoin, M., Johnson, S. R., Devries, A. J. G., Mohades-Kassai, A. & Tiedje, T. Temperature Dependence of the Optical Absorption Edge in Indium Phosphide. *MRS Proceedings,* **421,** 367 (1996).
7. Gong, X. *et al.* High-detectivity polymer photodetectors with spectral response from 300 nm to 1450 nm. *Science* **325,** 1665–1667 (2009).
